# 3D printing-enhanced transcatheter closure of residual shunts post-ventricular septal perforation: multimodal imaging’s crucial role

**DOI:** 10.1007/s12928-024-01064-8

**Published:** 2024-11-12

**Authors:** Daisuke Hachinohe, Hidehiko Hara, Kenji Makino, Ryo Horita, Hidemasa Shitan, Keijiro Mitsube

**Affiliations:** 1 Cardiovascular Medicine, Sapporo Cardio Vascular Clinic, 8-1, Kita-49 Higashi-16, Higashiku, Sapporo, 007-0849 Japan; 2 Toho University Ohashi Medical Center, Tokyo, Japan; 3 Makino Clinic, Tokyo, Japan; 4 Cardiovascular Surgery, Sapporo Cardio Vascular Clinic, Sapporo, Japan

**Keywords:** Recent myocardial infarction, Ventricular septal perforation, Residual shunt, Transcatheter closure

## Text

**Fig. 1:**
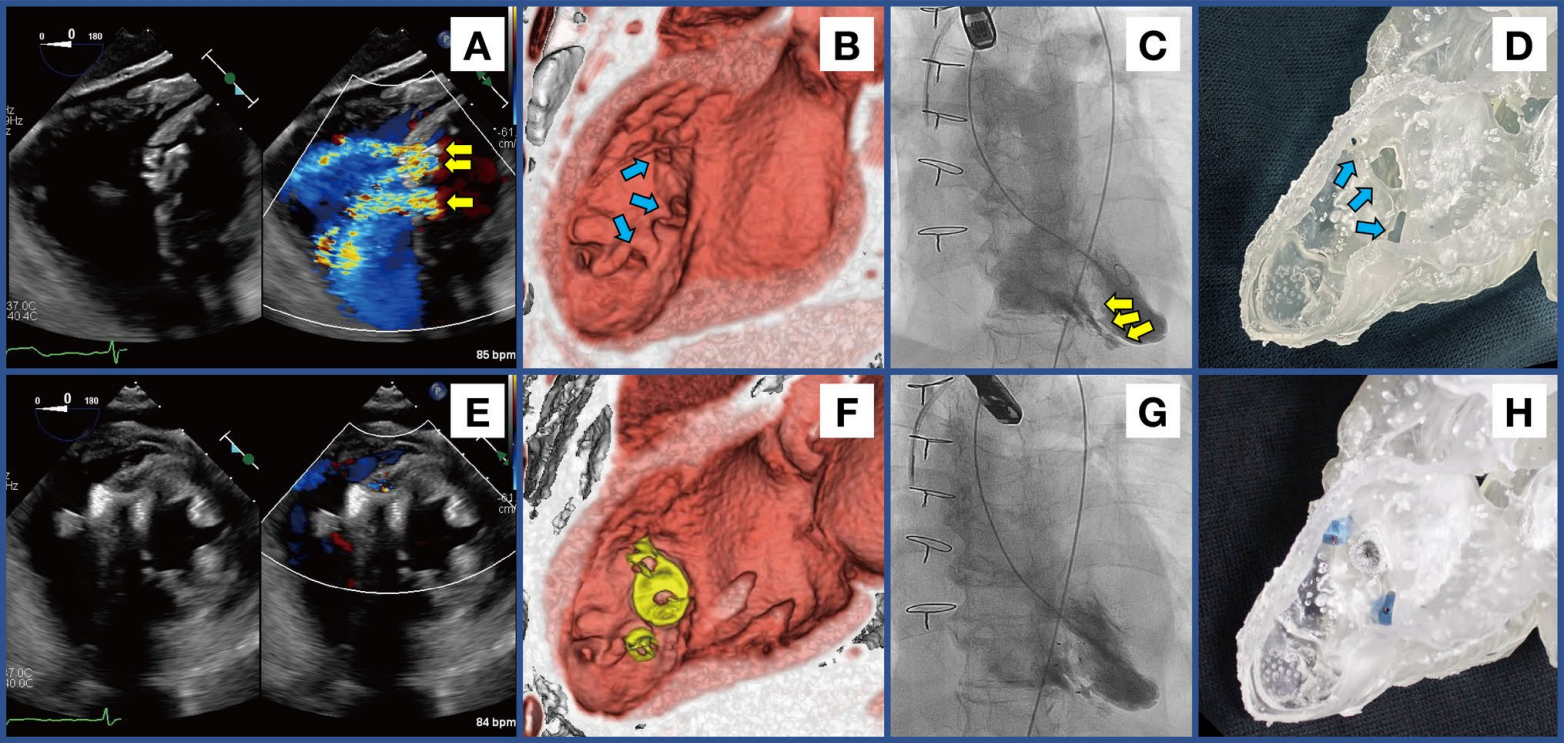
Transcatheter closure of porous residual shunts after ventricular septal perforation operation. **A** Preoperative transesophageal echocardiography (TEE), and **B** computed tomography (CT) shows porous residual shunts (arrows); **C** Left ventriculography (LVG) shows massive leaks (arrows) to right ventricle (RV) and pulmonary artery; **E** and **F** Well-occluded shunts by three plugs on TEE and CT; **G** LVG reveals minor leak to RV; **D** preoperative 3D model; **H** Fitting three plugs into the defects. *B, **D**, **F**, and **H** are images observed from almost the same angle on the left ventricular side

A woman in her 70 s was referred to our hospital 3 weeks after myocardial infarction. Transthoracic echocardiography (TTE) revealed a ventricular septal perforation and conservative management was initiated with intra-aortic balloon pumping assistance. Despite this, she developed shock and multiorgan failure. The surgical closure of the ventricular septal perforation was performed the following day. Postoperatively, residual shunts were noted around the patch, with a Qp/Qs ratio of 1.24 on TTE, which gradually worsened. Six weeks after surgery, the Qp/Qs ratio increased to 2.30, accompanied by worsening tricuspid regurgitation (TR). Transesophageal echocardiography (TEE) (Fig. 1A) and computed tomography (CT) (Fig. 1B/Supplementary Video 1) identified three significant shunts. The patient was in heart failure and while reoperation was considered, her severe frailty led to a refusal of another open-chest surgery. Consequently, transcatheter closure of the multiple residual defects was performed with the approval of our institution’s ethics committee. A wire was advanced through each shunt from the left ventricular side, followed by a pull-through from the femoral vein, and the shunts were occluded using two AMPLATZERTM Duct Occluders (ADO) II 03–04 and ADO I 14–12 (Abbott, Chicago, Illinois, USA) (Fig. 1C and G). TEE and CT confirmed successful occlusion of the three major shunts (Fig. 1E and F). Although a minor shunt remained, the Qp/Qs ratio improved to 1.51 and TR showed improvement, leading to clinical improvement. At the 1 year follow-up, there were no events such as heart failure recurrence, and TTE showed no residual shunt.

For this case, a preoperative 3D-printed model was created, which allowed us to study and select appropriate devices for the defect closures (Fig. 1D and H / Supplementary Video 2).

This represents the first report of transcatheter closure of three or more residual shunts following ventricular septal perforation repair. The repair of ventricular septal perforation in the acute phase can be challenging due to the fragility of infarcted myocardial tissue, often resulting in residual shunts [1]. While surgical repair is the standard approach, a 3D-printed model allows preoperative simulation of device selection and sizing. This is valuable as residual shunts in post-ventricular septal perforation vary and the model improves anatomical understanding, ensuring a safer and effective procedure.

## Supplementary Information

Below is the link to the electronic supplementary material.

Supplementary file1 (MP4 16523 KB)

Supplementary file2 (MP4 8902 KB)

## Data Availability

The data of this study are available upon reasonable request.
